# Understanding Motherhood After Stroke Occurring During Pregnancy, Childbirth, or the Postpartum Period: An Exploratory Mixed-Methods Study

**DOI:** 10.3390/healthcare14182967

**Published:** 2026-09-11

**Authors:** Cristina Gómez-Calero, Rebeca Martín-Peralvo, Miguel Brea-Rivero, Juan C. Pacho-Hernández, Olga I. Fernández-Rodríguez

**Affiliations:** 1 Department of Physiotherapy, Occupational Therapy, Rehabilitation and Physical Medicine, Health Sciences Faculty, Universidad Rey Juan Carlos, 28922 Madrid, Spain; cristina.gomez@urjc.es; 2 Gómez Ulla Central Defense Hospital, 28047 Madrid, Spain; rmarpe8@mde.es; 3 Department of Research and Psychology in Education, Universidad Complutense de Madrid, 28040 Madrid, Spain; jpacho@ucm.es; 4 INPRO Research Group, European University Miguel de Cervantes, 47012 Valladolid, Spain; oifernandez@uemc.es

**Keywords:** stroke, motherhood, pregnancy, postpartum period, maternal role, childcare, occupational performance, quality of life, rehabilitation, mixed methods

## Abstract

**Background/Objectives**: Stroke during pregnancy, childbirth, or the postpartum period is uncommon but may substantially affect women’s everyday functioning and maternal roles. This study aimed to characterize functional independence, occupational performance and satisfaction, and quality of life in women who had a stroke during these periods and to explore their lived experiences of motherhood. **Methods**: An exploratory mixed-methods study used sequential complementary quantitative and qualitative components. Eleven women completed quantitative assessments of functional independence, occupational performance and satisfaction, and quality of life. Six women participated in semi-structured interviews. Qualitative data were analyzed thematically by three researchers, and findings were integrated during interpretation. **Results**: Participants showed relatively high functional independence, with a median Functional Independence Measure total score of 111 (interquartile range = 8). Qualitative findings revealed disruption and gradual redefinition of the maternal role, difficulties in mother–child bonding and childcare, physical, cognitive, and emotional sequelae, reliance on family support, and perceived gaps between stroke-related care and motherhood-related needs. Integration of the findings showed that relatively preserved basic independence could coexist with substantial difficulties in maternal activities, while occupational priorities frequently related to childcare and family life. **Conclusions**: Motherhood after stroke involves challenges that may not be adequately captured by general measures of independence. The findings identify meaningful maternal occupations and the integration of stroke-related and motherhood-related needs as areas warranting further investigation within person-centered rehabilitation.

## 1. Introduction

Stroke is one of the leading causes of death and disability worldwide and places a heavy burden on those affected, their families and society [1]. Ischemic stroke is the most common stroke subtype globally, accounting for approximately 65.3% of incident strokes in 2021 [2]. Furthermore, stroke is associated with significant physical, cognitive, and emotional consequences that can affect independence, participation in everyday activities and social roles, and quality of life [3,4].

In the context of maternal health, stroke during pregnancy and the postpartum period is a rare but clinically serious complication. A systematic review and meta-analysis estimated a pooled crude rate of pregnancy-related stroke of 30.0 cases per 100,000 pregnancies [5]. Estimates vary across populations and methodologies; a study combining Taiwanese data with previous reports estimated an average incidence of 21.3 cases per 100,000 deliveries [6], while a large US analysis found that the incidence of acute stroke increased from 29.8 to 33.0 per 100,000 pregnancy-related hospitalizations between 2007 and 2015 when transient ischemic attacks and pregnancy-specific diagnostic codes were excluded [7]. Despite its low incidence, stroke associated with pregnancy is a significant cause of maternal morbidity and mortality [8].

Pregnancy and the postpartum period are associated with increased vulnerability to cerebrovascular events due to physiological, hemodynamic and procoagulant changes associated with pregnancy [9,10]. The risk is particularly elevated during the peripartum and postpartum periods [5], with pre-eclampsia and eclampsia being common causes of hemorrhagic strokes, while ischemic strokes are more frequently associated with cardioembolic events [6,8].

Although stroke recurrence during a subsequent pregnancy appears uncommon, women with a previous pregnancy-associated stroke may require individualized specialist follow-up because recurrence and other pregnancy complications remain clinically relevant [11,12]. Maternal stroke is therefore not only an acute event but also a condition with medium and long-term consequences that affect multiple aspects of a woman’s life.

Beyond its clinical consequences, stroke in the context of motherhood can profoundly disrupt daily life and the ability to fulfill meaningful roles. Stroke-related sequelae can interfere with a woman’s ability to care for herself and her child, as well as with the development of her maternal role [13,14].

Stroke can disrupt interacting motor, sensory, cognitive, communicative, emotional, and social functions that support everyday activities and meaningful life roles [4]. The consequences of these impairments depend not only on the functions affected but also on the specific demands of the activities and roles that the individual needs to perform. Motherhood is a particularly complex role because childcare requires the integration of physical abilities, attention, planning, problem-solving, communication, emotional regulation, safety monitoring, and adaptation to the child’s changing needs. Consequently, relatively preserved independence in basic activities may coexist with substantial difficulties in fulfilling maternal responsibilities. Qualitative evidence from younger stroke survivors who are parents shows that stroke-related impairments can disrupt parental identity, relationships, and parenting roles, with their impact influenced by the type and severity of impairments and the child’s age [13]. Studies in younger women have likewise described physical, psychosocial, and occupational consequences, including changes in life roles, work, self-care, and support needs [14].

However, despite these advances, the literature has focused primarily on epidemiological, etiological and clinical aspects, and there is limited understanding of the subjective experience of motherhood following a stroke. Although some qualitative research has explored the lived experiences of having a stroke among young people and parents, highlighting its impact on life roles and parental identity [13,14], there are few studies that specifically examine how women experience and reconstruct their role as mothers following a stroke. This limitation hinders the development of interventions tailored to their needs and focused on meaningful aspects of women’s lives, including motherhood. Given the heterogeneity of post-stroke impairment profiles and the diversity and complexity of maternal activities and responsibilities, qualitative investigation of women’s lived experiences is particularly valuable for identifying consequences and needs that may not be adequately captured by standardized functional measures [13,14].

Furthermore, some studies have shown that current models of post-stroke rehabilitation do not specifically address certain needs of young women, including psychosocial aspects and those related to life roles, which reinforces the need for further research focused on the maternal experience [15].

In this context, mixed-methods studies allow for the integration of quantitative and qualitative data, providing a more comprehensive understanding of complex health-related phenomena [16]. This approach is particularly relevant to the study of motherhood after stroke, where standardized measures of functioning, occupational performance, and quality of life can be complemented by women’s accounts of how stroke affects the maternal role and everyday life.

Therefore, this exploratory mixed-methods study aims to characterize functional independence, occupational performance and satisfaction, and quality of life in women who had a stroke during pregnancy, childbirth, or the postpartum period, and to explore their lived experiences of motherhood.

## 2. Materials and Methods

### 2.1. Study Design

A mixed-methods study was conducted using a sequential design comparing complementary quantitative and qualitative components. Quantitative and qualitative data were analyzed independently and subsequently integrated at the interpretation stage [16]. The quantitative component consisted of a cross-sectional study, while the qualitative component adopted a descriptive approach based on in-depth interviews. The quantitative data were collected and analyzed first, followed by qualitative data collection and analysis. The quantitative component was reported in accordance with the STROBE guidelines [17], and the qualitative component was reported in accordance with the SRQR standards [18] and the COREQ checklist [19].

### 2.2. Participants

Participants were recruited using a non-probability sampling strategy combining convenience and snowball sampling. Braining Mum, a Spanish association supporting mothers affected by acquired brain injury, facilitated initial access to some potentially eligible women, and additional women were identified through referrals from participants already enrolled in the study.

Women were eligible if they had had a stroke during pregnancy, childbirth, or the postpartum period. For eligibility purposes, the postpartum period corresponded to the immediate postpartum period described in the original study protocol as the postpartum “cuarentena” period. Eligibility for each study component required that, at the time of the corresponding data collection, the child born from that pregnancy was younger than 12 years. Participants were also required to have no pre-existing neurological disability, to be beyond the acute phase of recovery, to be able to understand the study procedures, and to provide written informed consent.

Eligibility was determined through participant self-report and confirmation of a previous medical diagnosis of stroke. Participants were asked to report whether the stroke occurred during pregnancy, childbirth, or the postpartum period, and eligibility was established according to these reports. Because the study focused on functional, occupational, and experiential aspects of motherhood, detailed clinical variables such as stroke subtype, lesion location, initial severity, acute treatments, rehabilitation history, residual neurological deficits, and obstetric complications were not systematically collected.

Because recruitment was conducted through convenience and snowball sampling and no complete sampling frame of potentially eligible women was available, the total size of the accessible population could not be determined. Seventeen women expressed interest in participating and were assessed for eligibility. Six did not meet the eligibility criteria: one had sustained a traumatic brain injury, one had sustained a brain injury before motherhood, three had children who exceeded the predefined age criterion, and one had acquired brain injury secondary to a brain tumor. The remaining 11 eligible women provided written informed consent and completed the quantitative assessment.

**Figure 1:**
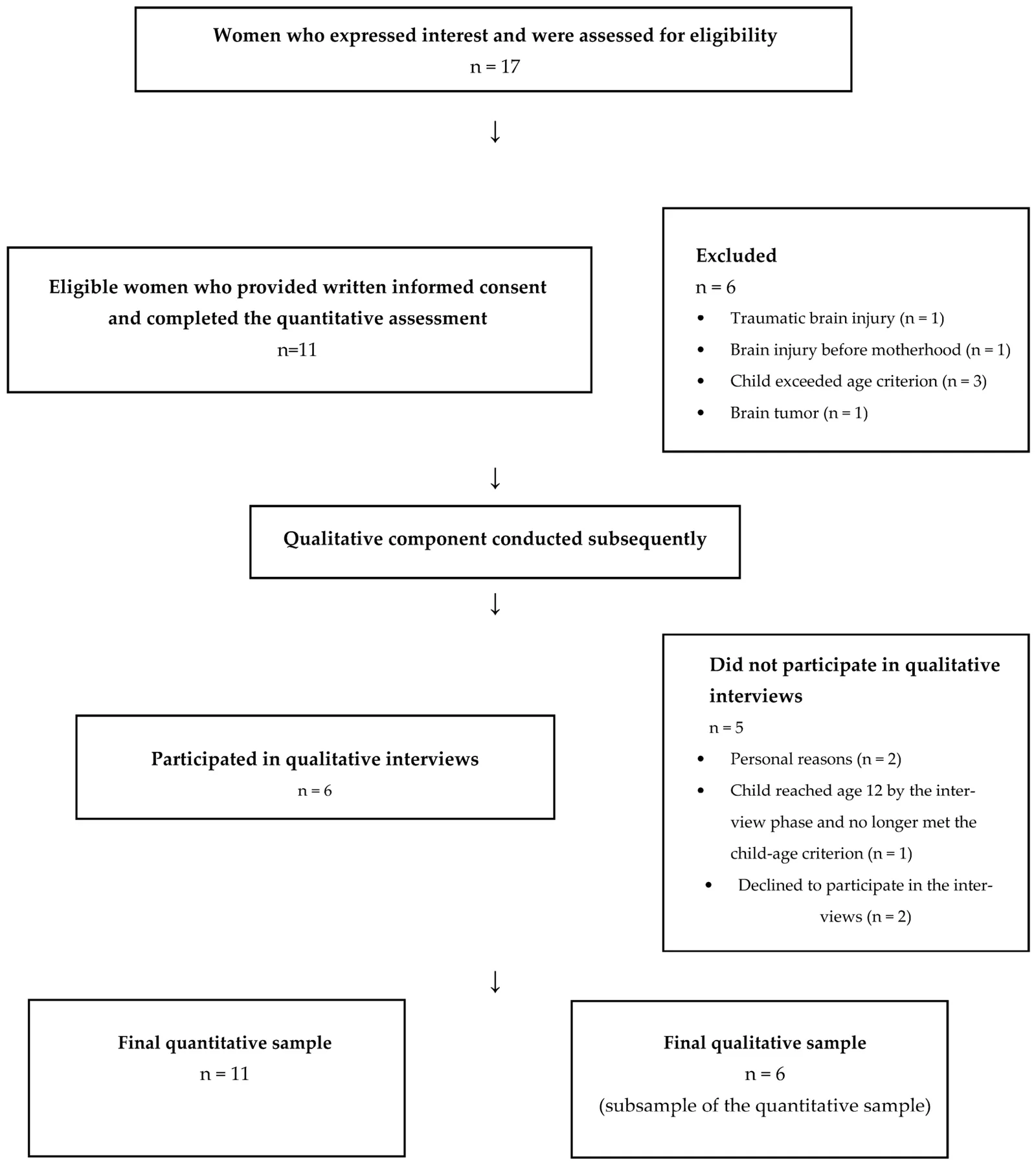
**Participant flow through the quantitative and qualitative components.** *Note.* All participants in the final quantitative sample had had a stroke.

The qualitative component was conducted subsequently. Of the 11 women who participated in the quantitative component, six subsequently took part in the semi-structured interviews. Five women did not participate in the qualitative component: two for personal reasons, one because her child had reached the age of 12 by the time of the interview phase and therefore no longer met the predefined child-age criterion, and two because they declined to participate in the interviews. Thus, the final quantitative sample comprised 11 women and the qualitative sample comprised a subsample of six of these participants. Participant flow through the quantitative and qualitative components is summarized in Figure 1.

### 2.3. Data Collection

Data collection was conducted remotely to facilitate the participation of women living in different regions of Spain. During the initial quantitative component, assessments were conducted by video call or telephone, according to participants’ communication needs and preferences. The subsequent qualitative interviews were conducted by videoconference using Microsoft Teams (Microsoft Corporation, Redmond, WA, USA). Data collection took place between 2022 and 2025.

#### 2.3.1. Sociodemographic and Clinical Variables

Sociodemographic and clinical data, including age, number of children, time elapsed since the stroke (months), birth order of the child, and timing of stroke onset (pregnancy, childbirth or postpartum), were collected using an ad hoc structured data collection form at the time of the quantitative assessment. For the qualitative subsample, the descriptive values presented in Table 1 were derived from the same data collected at the quantitative assessment for the six women who subsequently participated in the qualitative interviews. No additional clinical information regarding stroke subtype, lesion location, severity, treatment, rehabilitation history, residual neurological deficits, or obstetric complications was systematically collected.

#### 2.3.2. Quantitative Assessment

Functional independence was assessed using the Functional Independence Measure (FIM), an 18-item measure comprising 13 motor and five cognitive items [20]. The FIM has been widely used to assess functional independence in people following stroke and overall functional status [21]. Each item is scored from 1 to 7, with lower scores indicating greater dependence and higher scores indicating greater independence. The motor subscale ranges from 13 to 91 points and the cognitive subscale ranges from 5 to 35 points. The total score ranges from 18 to 126 points, with higher scores indicating greater functional independence. The FIM has demonstrated adequate reliability and validity [22,23]. In the Spanish context, satisfactory psychometric properties have also been reported for a Spanish cross-cultural adaptation of the combined FIM + FAM in people with stroke [24].

Occupational performance and participation, as well as satisfaction with occupational performance, were assessed using the Canadian Occupational Performance Measure (COPM), an instrument developed from person-centered practice and the Canadian Model of Occupational Performance [25]. The COPM enables identification of meaningful occupations and assessment of perceived performance and satisfaction on a scale of 1 to 10 [26]. The instrument has demonstrated validity and reliability in stroke patients [27], and a review of the literature has supported its clinical and research utility [28]. Furthermore, recent evidence regarding the measurement properties of the Spanish version of the COPM has shown satisfactory results in older adults undergoing inpatient rehabilitation, supporting the use of the Spanish version of the instrument [29].

Quality of life was assessed using a Spanish 64-item instrument designed to evaluate proxy-rated quality of life in people with acquired brain injury (CAVIDACE scale) [30]. The scale assesses eight dimensions (emotional well-being, interpersonal relationships, material well-being, personal development, physical well-being, self-determination, social inclusion and rights) and provides both an overall index and a profile of quality of life. The original proxy-report version of the CAVIDACE scale was used in the present study [30]. It was completed by a proxy respondent designated by the participant who knew her well. Raw scores for each dimension were converted into standardized scores. The standardized dimension scores were then summed, and the resulting composite score was converted into the Quality of Life Index and corresponding percentile according to the CAVIDACE scoring tables [30].

The quantitative assessment was completed in two sessions of approximately 45 min each, scheduled in advance with each participant. During the first session, sociodemographic data were collected and the FIM was administered. Before the second session, the CAVIDACE scale was sent by email to the proxy respondent designated by the participant for completion. The COPM was administered during the second session.

### 2.4. Sample Size

The sample size calculation for the quantitative component used an estimated population of approximately 8000 women with acquired brain injury in the Community of Madrid according to the 2008 Spanish Survey on Disability, Personal Autonomy and Dependency Situations (EDAD) [31]. This broader epidemiological population was used for the sample size calculation because a specific population estimate for women who had a stroke during pregnancy, childbirth, or the postpartum period was not available. The Community of Madrid was selected as the geographical reference because Braining Mum, the association that facilitated initial access to potentially eligible participants, is based in this region.

The calculation was based on the FIM. An expected standard deviation of 27.59 points was derived from the total FIM score reported for women with ischemic stroke by Senda et al. [32]. Using a 95% confidence level and a desired precision of ±15 FIM points, the calculation yielded a minimum sample size of 13 participants.

However, due to the low prevalence of stroke during pregnancy, childbirth and the postpartum period, together with the highly specific eligibility criteria and limited accessibility of this population, the final sample comprised 11 participants. Thus, the final sample was slightly below the prespecified sample size estimate.

No formal sample size calculation was performed for the qualitative component. The qualitative sample comprised the six participants from the quantitative sample who subsequently participated in the semi-structured interviews.

### 2.5. Qualitative Data Collection

The interviews were conducted by the first author, who has clinical experience in neurological rehabilitation and previous training in qualitative interviewing. At the time of the study, the interviewer was not involved in the participants’ clinical care. In-depth semi-structured interviews were conducted by videoconference using Microsoft Teams to explore motherhood following stroke and obtain detailed accounts of participants’ experiences, perceptions and interpretations. All interviews were video-recorded and subsequently transcribed verbatim for analysis. Illustrative quotations included in the manuscript were translated from Spanish into English by one of the authors who was involved in the qualitative interviews and in the analytic triangulation.

The qualitative component was designed as a descriptive qualitative study, an approach aimed at providing a comprehensive account of participants’ experiences while remaining close to their own descriptions of the phenomenon [33].

Interviews lasted approximately 60 to 90 min, allowing participants to provide detailed accounts of their experiences of stroke, motherhood, childcare, recovery, and support needs.

### 2.6. Data Analysis

#### 2.6.1. Quantitative Analysis

Quantitative analyses were performed using IBM SPSS Statistics, version 27 (IBM Corp., Armonk, NY, USA). Participant characteristics and quantitative outcomes were summarized descriptively. Continuous variables were reported using the mean and standard deviation and/or the median and interquartile range, as appropriate. Categorical variables were described using frequencies and percentages. Given the small overall sample and the very small and unequal stroke-onset subgroups, no inferential between-group comparisons were performed. FIM items and subscales, CAVIDACE dimensions, and the distribution of COPM occupational priorities and occupational areas were summarized descriptively.

#### 2.6.2. Qualitative Analysis

A thematic analysis [34] was carried out using MAXQDA 2020 (VERBI Software GmbH, Berlin, Germany). The analytical process was conducted through triangulation by three researchers and followed a hybrid approach, combining both deductive and inductive elements. In the first phase, initial categories aligned with the study’s objectives were defined by consensus. Subsequently, each researcher carried out independent coding, generating codes within those categories. In a second phase, the codes were compared and discussed among the three researchers and refined by consensus. During this process, overlapping or insufficiently supported codes were merged, revised, or removed; the initial categories were reorganized where necessary; and additional sub-themes were developed inductively when patterns not captured by the initial framework were identified in the data. This process enabled the construction of a final hierarchical system of themes and sub-themes. Reflexivity was addressed through regular discussions among the research team during coding and theme development. The involvement of researchers from different professional backgrounds facilitated critical examination of interpretations and helped challenge individual assumptions throughout the analytical process.

Given the rarity of stroke occurring during pregnancy, childbirth, or the postpartum period, together with the specific focus of the study on motherhood after stroke, the qualitative sample was considered to provide sufficient information power for an exploratory investigation. The aim was not to achieve statistical representation but to obtain rich and detailed accounts of participants’ experiences. Nevertheless, the findings should be considered exploratory and interpreted with appropriate caution.

### 2.7. Integration of Data

Quantitative and qualitative data were integrated during the interpretation phase by comparing the findings from both approaches, with the aim of identifying areas of convergence, complementarity, and divergence [16]. Integration focused on how the quantitative findings on functional independence, occupational performance and satisfaction, and quality of life related to the qualitative themes concerning the experience of motherhood and everyday life after stroke. The two components retained their analytical independence, and integration occurred at the level of interpretation rather than by combining the datasets into a single analysis.

### 2.8. Ethical Considerations

The study was approved by the Ethics Committee of the first author’s institution. A first favorable opinion was obtained on 25 February 2022 (internal registration no. 2710202120621), followed by a subsequent favorable opinion on 9 November 2022 (internal registration no. ENM 206/210509202218422). The principles of the Declaration of Helsinki and applicable Spanish legislation were followed.

All participants signed a written informed consent form before participating. Participants who took part in the qualitative component provided additional written informed consent before the interview. Participant data were pseudonymized using study codes to protect confidentiality. Video recordings of the qualitative interviews were deleted after verbatim transcription, and only the pseudonymized transcripts were retained for analysis.

## 3. Results

### 3.1. Sample Characteristics

A total of 11 women, all of whom had had a stroke, were included in the final quantitative sample. Of these 11 women, six also participated in the qualitative component: three had had a stroke during pregnancy, one during childbirth, and two during the postpartum period. The characteristics of the quantitative and qualitative samples are presented in Table 1.

### 3.2. Quantitative Results

#### Descriptive Quantitative Findings

Overall, participants showed relatively high levels of functional independence. The median FIM total score for the quantitative sample was 111 (IQR = 8). Descriptively, motor subscale scores were closer to the maximum score than cognitive subscale scores (median = 85 of 91 and 26 of 35, respectively; IQR = 10 for both). The median CAVIDACE Quality of Life Index was 109 (IQR = 22). Regarding occupational performance, the median COPM mean performance score was 6.0 (IQR = 2.0), whereas the median mean satisfaction score was 8.6 (IQR = 2.4). Detailed descriptive profiles for the FIM and CAVIDACE dimensions are provided in Supplementary Tables S1 and S2, respectively.

The COPM identified 55 occupational priorities representing 28 distinct activities. Many of the selected priorities were related to childcare and family life. Going to the park with the children was the most frequently identified activity (*n* = 7), followed by helping children with homework (*n* = 4). Holding or carrying the child, taking children to school or nursery, engaging in physical games, and travelling with the family were each identified three times. Other childcare-related priorities included dressing or feeding children, reading stories, attending extracurricular activities, sleeping with the child, and taking the child to medical appointments. The complete distribution of occupational priorities is provided in Supplementary Table S3.

### 3.3. Qualitative Results

The qualitative analysis identified five themes that captured the women’s experiences of motherhood following stroke: (1) stroke during motherhood; (2) stroke sequelae; (3) the experience of being a mother after stroke; (4) childcare after stroke; and (5) support and the healthcare system. The themes and corresponding subthemes are summarized in Table 2.

#### 3.3.1. Theme 1: Stroke During Motherhood

##### Subtheme 1: Childbirth Following Stroke During Pregnancy

For participants whose stroke occurred during pregnancy, childbirth was overshadowed by the neurological condition. The participants described a disconnect between their expectations of motherhood and their actual experience of childbirth.

This disruption was reflected in the loss of an anticipated moment, as captured in the quote “they stole my moment” (P3). The difficulty in processing what had happened was associated with cognitive and emotional disconnection, as reflected in: “my mind wasn’t there at the time” (P3), “I can’t recall that time in hospital” (P6).

A lack of emotional response to the birth was also a recurring theme: “I didn’t react like other mums” (P3), or “Oh, right” (P3), highlighting a disconnect between their lived experience and their expectations of motherhood. However, in some cases, conflicting emotions coexisted: “I was really scared… but when I heard her cry, I was moved” (P1).

Overall, childbirth proved to be a fragmented experience, shaped by altered consciousness and the difficulty of integrating the birth into one’s own experience.

##### Subtheme 2: Stroke Occurring During Childbirth

For the participant whose stroke occurred during childbirth, the experience was characterized by the severity of the event and a profound disruption of the birth experience. The birth was experienced as a critical event: “They had to make an incision to get the baby out” (P4), with survival being the priority.

Subsequently, a process of meaning-making emerged, including the attribution of blame: “I blamed XX (the baby)… I blamed everyone” (P4), reflecting the difficulty in coming to terms with an abrupt and traumatic experience.

##### Subtheme 3: Stroke Occurring During the Postpartum Period

Stroke occurring during the postpartum period was characterized by fragmented experiences and delayed awareness. Participants described experiences ranging from a complete lack of memory—“I don’t remember any of that” (P2)—to vague memories—“my memory of it is very hazy” (P5).

Experiences of cognitive disorganization were also reported: “I have flashes… I started talking nonsense” (P5). Emotional distress was also described: “lots of anxiety attacks” (P2).

##### Subtheme 4: Consciousness During the Acute Phase

Altered level of consciousness was a recurring theme that affected both the experience of stroke and the perception of motherhood. Participants described periods of absent or fragmented awareness, including “I’m not conscious… you are not aware of anything” (P6). Other participants expressed this phase through experiences of unreality, “like a dream”, “like a horror film” (P2), or a sense of detachment, “my head was somewhere else” (P3).

In the most severe cases, there was a disruption in identity: “It wasn’t me… I didn’t recognize my children” (P4). This discontinuity also affected the awareness of motherhood: “I wasn’t really aware that I had brain damage and that I had two children” (P2), highlighting a rupture in the continuity of the self.

#### 3.3.2. Theme 2: Stroke Sequelae

##### Subtheme 1: Physical Sequelae

Physical limitations resulted in a significant loss of independence and had a major impact on daily life and motherhood. Some participants reported situations of extreme dependence: “I had a quadriplegia; I couldn’t do anything” (P2), “I was in a wheelchair” (P4).

These limitations resulted in specific difficulties with care: “Standing, I couldn’t pick the child up and walk” (P3), “I can’t walk up a hill” (P3), as well as dependence for basic activities: “they bathed me”, “diaper” (P2). Fatigue was also a significant factor: “I ended up exhausted to speak” (P1).

##### Subtheme 2: Cognitive Sequelae

Cognitive complications were particularly limiting, affecting comprehension, communication and organization. Participants expressed general difficulties, such as “my brain couldn’t take it anymore” (P2) and “I can’t make sense of anything” (P1), as well as specific impairments, such as “dysphasia and dyscalculia” (P5).

These limitations had a direct impact on parenting: “I’m very disorganized” (P3), especially in overwhelming situations: “if I’m really overwhelmed, I can’t function” (P3). Furthermore, they felt invisible—“people don’t see that” (P3)—which only added to the experience of being misunderstood.

##### Subtheme 3: Psychological Sequelae

The psychological impact was profound and persistent. Participants reported thoughts of death, “I’d be much better off dead” (P2), feelings of worthlessness, “being useless” (P2), and a heavy mental burden, “my mind was going at 10,000 miles an hour” (P2).

Difficulties with acceptance and identity were also evident, “it hurts to accept that it has happened” (P4), “I’ve found it hard to look at myself in the mirror” (P3), along with experiences of feeling misunderstood, “I’ve felt misunderstood and I’ve cried out of helplessness” (P3).

##### Subtheme 4: Sensory and Invisible Sequelae

The invisible and sensory long-term effects limited their participation in everyday life. Participants described problems with stimuli, “problems with noises and lights” (P5), cognitive overload, “too much information for my head” (P3), and visual difficulties, “I couldn’t see the products” (P6). These limitations affected activities such as shopping or reading, “I couldn’t read” (P3), highlighting their functional impact.

#### 3.3.3. Theme 3: The Experience of Being a Mother After Stroke

##### Subtheme 1: Loss and Redefinition of the Maternal Role

Stroke was associated with disruption of the participants’ maternal role. They expressed, “I wasn’t a mother” (P2), “my role as a mother is lost” (P2), or that motherhood was purely biological: “I had the title… nothing more” (P2).

However, a gradual redefinition was observed: “until my mother left home, I didn’t fulfill the role as a mother” (P5), and a greater identification with the role over time: “that’s when I feel more like a mother” (P6). These accounts showed that the redefinition of their role was not immediate, but rather a gradual process closely linked to both their functional recovery and the level of support they received.

##### Subtheme 2: Mother–Child Bond

The bond was affected, particularly in the early stages, by experiences of rejection—“I didn’t hold my baby or cuddle him” (P4)—or by a lack of bonding—“there was no sense of excitement” (P3). Avoidance was also described: “I didn’t want them to bring them to me” (P2). However, the bond could be rebuilt: “It took me a long time to restore the bond” (P2).

#### 3.3.4. Theme 4: Childcare After Stroke

Difficulties with childcare were widespread and extended far beyond breastfeeding or supervision. Participants highlighted, “I couldn’t look after my daughter” (P5) and “I didn’t look after her during the first months” (P5), showing that other family members often assumed primary responsibility for childcare during the early stages.

Breastfeeding was particularly significant: “I couldn’t breastfeed” (P5), “I cried a lot… and I just couldn’t” (P3). Intense fears associated with the baby’s hygiene and safety were also described, “terrifying fear” (P5), “I didn’t feel confident enough to give him a bath” (P2), as well as the inability to perform basic tasks such as cleaning, bathing, dressing or changing the diaper. In some cases, even when the mother was involved in care, she did so under conditions of dependence or supervision, “always with people by my side” (P5).

These limitations also affected the mother’s ability to comfort and physically support her child: “I couldn’t pick the child up when he was crying” (P3), “it was killing me slowly” (P3). Thus, childcare was affected not only functionally but also emotionally.

#### 3.3.5. Theme 5: Support and the Healthcare System

##### Subtheme 1: Family Network and Partner

The family was the main source of support. Partners took on intensive caregiving roles: “24 h a day at home” (P2), “we are a team” (P5), “my partner is a 10” (P1). Family support helped sustain both recovery and childcare, particularly in the early stages when participants were most dependent.

However, tensions also arose: “I just hated my husband” (P3) or “my family didn’t understand me” (P3). Participants experienced their relationship with their partner shifting toward caregiving dynamics, while the family network, although essential, was also sometimes perceived as a source of conflict, misunderstanding or a loss of autonomy.

##### Subtheme 2: Professionals and the Healthcare System

Participants perceived a lack of integration between stroke-related care and motherhood. This concern extended beyond a lack of practical training. In their own accounts, participants described this perceived separation by stating that “Everything was attributed to brain injury… not to motherhood” (P6) and that “having a brain injury is not the same as having a child” (P2), highlighting that motherhood was not specifically taken into account in care delivery.

Participants also highlighted a lack of preparation: “Nobody prepares you for this” (P6), describing the challenge of coping simultaneously with the consequences of stroke and the demands of motherhood. They described failures in care delivery during the acute phase, “she has to go to hospital on her own” (P3), or delays in diagnosis (P2). In subsequent stages, participants highlighted the lack of realistic training, “With a baby doll… that’s not reality” (P6), and the absence of specific support, “there’s no one to teach you techniques” (P2).

In addition, some specific concerns emerged, such as the need for motherhood to be taken into account when assessing independence, the lack of information provided to families about cognitive sequelae, the need for more contact with the baby during hospitalization, and a demand for specialist units and specific support for childcare. Although some experiences were positive, such as feeling “supported” by professionals (P6), overall, the system was perceived as providing an insufficient response to provide integrated care that addresses the complexity of motherhood following stroke.

### 3.4. Integration of Quantitative and Qualitative Data

The integration of the quantitative and qualitative findings demonstrated areas of convergence and notable divergence. First, the FIM data reported levels approaching independence in various self-care activities, sphincter control, and transfers. However, the qualitative accounts showed that the most significant difficulties involved childcare, the mother–child bond, and the cognitive demands of motherhood rather than self-care. This divergence is particularly significant, as it suggests that a relatively well-preserved level of basic functional independence does not necessarily imply a sense of maternal competence.

Second, the descriptive FIM profile also suggested greater preservation of motor than cognitive functional independence, with motor subscale scores closer to the maximum score. Descriptive variability in cognitive items, particularly problem-solving and memory, was consistent with the qualitative findings regarding cognitive sequelae. The interviews revealed difficulties with attention, organization, multitasking, and cognitive overload, providing context for functional difficulties that may be less apparent from the relatively high total FIM score.

Third, the COPM findings showed that the prioritized occupations were frequently related to the maternal role and childcare, including going to the park, helping with homework, holding or carrying the child, taking them to school/nursery, engaging in physical games, and travelling with the family. These results converged directly with the qualitative themes about the loss and redefinition of the maternal role, specific difficulties in childcare, and the need for support to resume meaningful activities related to motherhood.

Finally, the quantitative findings reflected variability across participants in functional independence, occupational performance, and quality of life. The qualitative data further illustrated considerable variability in individual experiences, particularly regarding the mother–child bond, family support, psychological sequelae, and interactions with the healthcare system. Given the exploratory nature of the study and the small sample size, this variability could not be examined in greater depth. In this regard, overall measures of quality of life appear to capture only part of the complexity and variability of the maternal lived experience following stroke. Taken together, the integration of both approaches provided an understanding not only of functional and occupational limitations, but also of their meaning within motherhood. The findings suggest that motherhood following stroke may not be fully understood solely in terms of functional independence or overall quality of life but also in terms of the tension between retained basic autonomy, cognitive overload, a subjective sense of loss of the role, and the need for gradual reconstruction within a context of uneven support.

## 4. Discussion

This exploratory mixed-methods study provides an initial overview of the impact of stroke on motherhood, combining quantitative data on functional independence, occupational performance and participation, satisfaction with occupational performance, and quality of life with an in-depth exploration of women’s lived experiences. The findings showed that motherhood following stroke is a complex process, characterized by an initial interruption of the maternal role, the presence of persistent long-term effects, and a gradual redefinition within a context shaped by both social support and the limitations of the healthcare system. To the best of our knowledge, this is one of the first studies to specifically examine the experience of motherhood following stroke during pregnancy, childbirth, or the postpartum period using a mixed-methods approach, combining functional outcomes and subjective lived experiences.

The quantitative analysis provided a descriptive characterization of functional independence, occupational performance and satisfaction, and quality of life among women who had had a stroke during pregnancy, childbirth, or the postpartum period. Given the small sample size and exploratory nature of the study, these findings should be interpreted with appropriate caution.

Alongside these quantitative findings, qualitative findings highlighted marked variability in individual experiences, revealing the importance of integrating both approaches. Participants described profound changes in their experience of motherhood, particularly in the early stages following stroke, where altered consciousness, emotional detachment and functional limitations made it difficult to process the birth experience and to establish a bond with their child. These findings are consistent with previous evidence highlighting the impact of stroke on multiple dimensions of life, including emotional, cognitive and social aspects [4].

Furthermore, qualitative findings of this study are consistent with previous research showing that stroke may alter parental identity and the ability to perform caregiving roles, leading to changes in family dynamics and the relationship with children [13]. Our findings extend this evidence by focusing specifically on motherhood in the context of stroke occurring during pregnancy, childbirth and the postpartum period.

One of the most notable findings of the study is the mismatch between the relatively well-preserved levels of functional independence in essential activities of daily living, measured by the FIM, and the perception of being unable to fulfill the maternal role. While participants demonstrated autonomy in tasks such as eating or basic mobility, they indicated substantial difficulties in activities related to parenting, such as physically handling their child or managing multiple demands simultaneously. This divergence suggests that general measures of functional independence may not adequately capture the specific demands of fulfilling the maternal role, particularly those related to cognitive and emotional burden.

The COPM results further indicate that the occupations prioritized by participants were closely linked to the maternal role. This finding is consistent with previous qualitative evidence showing that stroke can disrupt parental identity and parenting roles, as well as broader everyday roles and occupations in younger women [13,14]. Another important finding was the perceived lack of integration by demonstrating that general functional limitations do not adequately reflect the specific demands of performing the maternal role.

Another important aspect is the impact of cognitive and psychological sequelae, which emerged as particularly limiting factors in participants’ qualitative accounts. These difficulties, often invisible, affect both occupational performance and the perception of maternal competence. This finding is consistent with the literature, which highlights the importance of psychosocial factors in recovery following a stroke [4].

Childcare appeared to be one of the most severely affected areas, becoming a key source of difficulty. The need for external support, while functionally necessary, contributed in many cases to the loss or disruption of the maternal role. Participants identified childcare-related difficulties as a major challenge, suggesting that this area may warrant further investigation in future intervention studies.

With regard to social support, the family played a key role in the recovery process, although changes in family dynamics and tensions within the partner’s relationship were also evident. These findings reflect the complexity of family adaptation dynamics following a neurological event and suggest that the potential role of the immediate social environment in rehabilitation warrants further investigation.

Furthermore, another important finding was the perceived lack of integration within the healthcare system between stroke-related care and the specific demands of motherhood. Participants described fragmented care in which stroke-related needs and parenting were addressed separately. This lived experience aligns with findings from recent studies suggesting that post-stroke rehabilitation programs may not adequately address the specific needs of young women, particularly in terms of physical, psychological and life role-related aspects, suggesting the potential value of personalized, experience-centered approaches that should be explored in future research [15].

An additional consideration when interpreting these findings is the heterogeneity of stroke presentations. Ischemic stroke and intracerebral hemorrhage may differ substantially in terms of underlying mechanisms, functional consequences, recovery trajectories, and rehabilitation requirements. Although the present study explored motherhood following stroke as a shared lived experience, it is possible that the challenges, support needs, occupational priorities, and rehabilitation requirements reported by participants may vary according to stroke subtype and associated clinical characteristics. Therefore, the implications of the present findings should be interpreted with caution and should not be assumed to apply uniformly across all stroke presentations. Future research should specifically examine whether motherhood experiences, caregiving challenges, occupational priorities, psychosocial adjustment, and rehabilitation needs differ according to stroke subtype and associated clinical characteristics.

From a mixed-methods perspective, the quantitative and qualitative components provided complementary information. The quantitative measures characterized functional independence, occupational performance and satisfaction, and quality of life, while the qualitative findings illuminated how functional, cognitive, emotional, relational, and caregiving challenges were experienced within motherhood.

This study has several limitations. First, the overall sample was small and slightly below the initial sample size estimate of 13 participants, and the quantitative stroke-onset subgroups were very small and unequal, particularly the childbirth subgroup, which included only two participants. These characteristics precluded meaningful inferential comparisons between stroke-onset groups. The quantitative findings should therefore be interpreted descriptively and should not be generalized beyond the present sample.

Second, participants were recruited using non-probability convenience and snowball sampling, with initial access to some potentially eligible women facilitated by Braining Mum. This recruitment strategy may have introduced selection bias and limits the generalizability of the findings to the wider population of women who have a stroke in relation to motherhood. Recruitment was further constrained by the rarity of the target population and the specific eligibility criteria. Although all 11 eligible women completed the quantitative assessment, only six participated in the subsequent qualitative component. The qualitative subsample was also small and unevenly distributed across stroke-onset periods (pregnancy, *n* = 3; childbirth, *n* = 1; postpartum, *n* = 2). Therefore, timing-specific qualitative observations, particularly those concerning childbirth, should be interpreted cautiously and should not be considered representative of women within each stroke-onset period. Although the interviews provided rich experiential accounts, the small qualitative sample may not have captured the full diversity of experiences of motherhood following stroke. Consequently, the qualitative findings should be considered exploratory and interpreted with appropriate caution.

Third, recall bias may also have affected the findings. Participants reported retrospectively on experiences related to stroke, childbirth, rehabilitation, and motherhood, and the time elapsed since stroke varied across participants. As a result, memories of acute events and early maternal experiences may have been influenced by later recovery, adaptation processes, and subsequent experiences, potentially affecting the accuracy and interpretation of some accounts.

Finally, detailed clinical and obstetric variables were not systematically collected. Information regarding stroke subtype, lesion location, initial stroke severity, acute management, rehabilitation history, major residual neurological deficits, and pregnancy- or childbirth-related complications was therefore unavailable. These factors may substantially influence functional outcomes, quality of life, and maternal experiences. Consequently, variability across participants could not be explored in relation to these characteristics, limiting the interpretation of potential sources of heterogeneity within the sample. In particular, the absence of systematic information regarding stroke subtype prevented exploration of potential differences between women with ischemic stroke and those with intracerebral hemorrhage or other cerebrovascular events. Therefore, it was not possible to determine whether maternal experiences, occupational priorities, support needs, or rehabilitation implications differed according to stroke type.

Despite these limitations, the qualitative component provides detailed insight into an understudied phenomenon and, together with the quantitative findings, identifies aspects of motherhood that may not be adequately represented by general measures of functional independence and quality of life.

From a clinical perspective, the findings generate hypotheses regarding the potential value of more integrated approaches to care, which require further investigation in larger studies, particularly in relation to the preparation, guidance, and support provided to women during rehabilitation. The findings suggest that the involvement of family members and partners may be relevant and warrants further investigation. Finally, participants’ accounts identified attention to meaningful maternal occupations as a potentially relevant area for future rehabilitation research. The potential contribution of occupational therapy and tools such as the COPM to identifying these priorities warrants further evaluation in larger studies.

This study highlights several directions for future research that may help deepen our understanding of motherhood following stroke. First, future studies should include larger samples to provide more precise quantitative estimates. Such studies could also explore whether maternal experiences, functional outcomes, support needs, and rehabilitation priorities vary according to both the timing of stroke onset and stroke subtype, including ischemic and intracerebral hemorrhagic stroke. Longitudinal research could further examine how the maternal role, childcare participation, and the mother–child relationship evolves throughout recovery. In addition, future studies could evaluate interventions specifically addressing the occupational, cognitive, emotional, and support needs associated with motherhood after stroke.

## 5. Conclusions

Motherhood following stroke is a complex experience, characterized by an initial disruption of the maternal role, difficulties in bonding with the child, and significant limitations in care during the early stages. As the recovery process progresses, women gradually redefine their identity as mothers, influenced by their functional progress, cognitive and emotional sequelae, and the social support they receive. The qualitative findings further showed that childcare difficulties, dependence on others, changes in family relationships, and perceived gaps between stroke-related care and the specific needs associated with motherhood shaped this process of adaptation.

The quantitative results showed relatively well-preserved levels of functional independence. However, integration with qualitative data revealed that these measures do not fully capture the complexity of motherhood after stroke, particularly in relation to the specific demands of childcare, the impact of cognitive sequelae, and the challenges associated with performing the maternal role. Occupational priorities identified by the women were frequently related to childcare and family life, suggesting that meaningful maternal occupations may represent a relevant area for future rehabilitation research and service development.

The findings of this exploratory study suggest that motherhood following stroke may not be fully understood solely through general functional indicators; rather, it requires consideration of how these limitations affect the maternal role. The combination of quantitative and qualitative methods provided a broader and more nuanced understanding of the phenomenon, suggesting that integrated approaches addressing both stroke-related and motherhood-related experiences represent a potentially relevant area for future research and clinical development.

## Figures and Tables

**Table 1:** Characteristics of the quantitative and qualitative samples.

Characteristic	Quantitative Sample (*n* = 11)	Qualitative Sample (*n* = 6)
Age, years, mean ± SD	39.45 ± 6.70	40.50 ± 1.52
Time since stroke, months, mean ± SD	46.18 ± 19.91	55.50 ± 20.00
Number of children, *n* (%)		
1 child	7 (63.6)	3 (50.0)
2 children	4 (36.4)	3 (50.0)
Stroke associated with first child, *n* (%)	7 (63.6)	3 (50.0)
Stroke during pregnancy, *n* (%)	5 (45.5)	3 (50.0)
Stroke during childbirth, *n* (%)	2 (18.2)	1 (16.7)
Stroke during postpartum period, *n* (%)	4 (36.4)	2 (33.3)

*Note*. For both samples, time since stroke refers to the interval recorded at the quantitative assessment. Values for the qualitative sample correspond to the six women who subsequently participated in the qualitative interviews. SD: standard deviation.

**Table 2:** Themes and subthemes identified in the qualitative analysis.

Theme	Subtheme
1. Stroke during motherhood	1.1. Childbirth following stroke during pregnancy
	1.2. Stroke occurring during childbirth
	1.3. Stroke occurring during the postpartum period
	1.4. Consciousness during the acute phase
2. Stroke sequelae	2.1. Physical sequelae
	2.2. Cognitive sequelae
	2.3. Psychological sequelae
	2.4. Sensory and invisible sequelae
3. The experience of being a mother after stroke	3.1. Loss and redefinition of the maternal role
	3.2. Mother-child bond
4. Childcare after stroke	—
5. Support and the healthcare system	5.1. Family network and partner
	5.2. Professionals and the healthcare system

*Note*. — = no subtheme identified.

## Data Availability

The materials and analysis code for this study are not available in any repository; however, we will make our data accessible upon request to the corresponding author.

## Supplementary Materials

The following supporting information can be downloaded at: https://www.mdpi.com/article/10.3390/healthcare14182967/s1. Table S1: Detailed descriptive profile of the Functional Independence Measure (FIM) in the quantitative sample; Table S2: Descriptive profile of CAVIDACE dimensions in the quantitative sample; Table S3: Occupational priorities identified using the Canadian Occupational Performance Measure (COPM).
