# Supplementary material for: Understanding Motherhood After Stroke Occurring During Pregnancy, Childbirth, or the Postpartum Period: An Exploratory Mixed-Methods Study
Source: Healthcare (Basel). 2026 Sep 11;14(18):2967. doi: 10.3390/healthcare14182967 (PMC13606800; doi:10.3390/healthcare14182967)
Supplement: Supplementary file 1 [file healthcare-14-02967-s001.zip › healthcare-4414482-supplementary.pdf]

Supplementary Materials

Table S1. Detailed descriptive profile of the Functional Independence Measure (FIM) in the quantitative sample (n = 11).

FIM item/subscale	Mean ± SD	Median (IQR)	Range
Eating	6.27 ± 1.19	7 (1)	4-7
Grooming	6.27 ± 1.01	7 (1)	4-7
Bathing	6.27 ± 0.90	7 (2)	5-7
Dressing - upper body	6.64 ± 0.67	7 (1)	5-7
Dressing - lower body	6.73 ± 0.65	7 (0)	5-7
Toileting	6.73 ± 0.65	7 (0)	5-7
Bladder management	7.00 ± 0.00	7 (0)	7-7
Bowel management	7.00 ± 0.00	7 (0)	7-7
Bed/chair/wheelchair transfer	7.00 ± 0.00	7 (0)	7-7
Toilet transfer	7.00 ± 0.00	7 (0)	7-7
Tub/shower transfer	6.91 ± 0.30	7 (0)	6-7
Walk/wheelchair	6.18 ± 1.47	7 (1)	2-7
Stairs	5.73 ± 1.62	7 (3)	3-7
Comprehension	6.36 ± 1.03	7 (1)	4-7
Expression	5.82 ± 1.54	7 (3)	3-7
Social interaction	5.27 ± 1.62	5 (3)	3-7
Problem solving	4.00 ± 1.61	4 (2)	2-7
Memory	4.55 ± 1.97	4 (4)	2-7
**Motor subscale**	85.73 ± 5.22	85 (10)	77-91
**Cognitive subscale**	26.00 ± 5.24	26 (10)	19-35
**Total FIM score**	111.73 ± 5.52	111 (8)	104-123

Note. FIM item scores range from 1 to 7, with higher scores indicating greater independence. The motor subscale ranges from 13 to 91, the cognitive subscale from 5 to 35, and the total score from 18 to 126. IQR = interquartile range; SD = standard deviation.

Table S2. Descriptive profile of CAVIDACE dimensions and summary scores in the quantitative sample (n = 11).

**CAVIDACE score**	**Mean ± SD**	**Median (IQR)**	**Range**
Emotional well-being	11.73 ± 2.57	12 (5)	8-16
Interpersonal relationships	12.55 ± 2.34	13 (5)	9-15
Material well-being	12.09 ± 2.30	13 (2)	7-15
Personal development	14.00 ± 2.32	14 (4)	9-17
Physical well-being	9.09 ± 4.87	11 (9)	3-15
Self-determination	12.55 ± 1.75	13 (3)	9-14
Social inclusion	12.09 ± 3.18	12 (5)	7-16
Rights	10.73 ± 2.80	10 (6)	7-14
Sum of standardized dimension scores	94.82 ± 12.54	90 (25)	82-118
**Quality of Life Index**	112.91 ± 10.88	109 (22)	102-133
Percentile	75.45 ± 16.82	72 (36)	55-99

Note. Dimension scores are standardized scores derived according to the CAVIDACE scoring procedure. The standardized dimension scores are summed and converted into the Quality of Life Index and corresponding percentile. IQR = interquartile range; SD = standard deviation.

Table S3. Occupational priorities identified using the Canadian Occupational Performance Measure (COPM) in the quantitative sample (n = 11).

Panel A. Distribution of occupational priorities identified through the COPM

**Occupational priority**	**n**	**Participants (%)**
Going to the park with one's children	7	63.6
Helping one's children with homework	4	36.4
Travelling as a family	3	27.3
Managing finances	3	27.3
Playing physical games	3	27.3
Taking children to school/nursery	3	27.3
Holding one's children	3	27.3
Grooming	2	18.2
Dressing one's children	2	18.2
Using public transport	2	18.2
Going for a walk as a family	2	18.2
Volunteering	2	18.2
Social activities and outings with one's children	2	18.2
Driving	2	18.2
Cooking	2	18.2
Reading storybooks	1	9.1
Feeding one's children	1	9.1
Completing studies	1	9.1
Children's extracurricular activities	1	9.1
Using the phone	1	9.1
Dancing	1	9.1
Shopping	1	9.1
Using email	1	9.1
Brushing children's hair	1	9.1
Doing household tasks	1	9.1
Going swimming with one's children	1	9.1
Sleeping with one's children	1	9.1
Taking one's children to the doctor	1	9.1

Note. Participants identified five occupational priorities each (55 priorities in total). Percentages are calculated using the quantitative sample (n = 11) as the denominator and therefore do not sum to 100%. Semantically equivalent labels for public transport were combined.

Panel B. Distribution of occupational priorities by COPM occupational area

Occupational area	n	Occupational priorities (%)
Self-care	12	21.8
Productivity	20	36.4
Leisure	23	41.8
**Total**	55	100.0

Note. Percentages are calculated using the total number of occupational priorities identified across participants (N = 55) as the denominator.
